# The Activation of Gluteal, Thigh, and Lower Back Muscles in Different Squat Variations Performed by Competitive Bodybuilders: Implications for Resistance Training

**DOI:** 10.3390/ijerph18020772

**Published:** 2021-01-18

**Authors:** Giuseppe Coratella, Gianpaolo Tornatore, Francesca Caccavale, Stefano Longo, Fabio Esposito, Emiliano Cè

**Affiliations:** 1 Department of Biomedical Sciences for Health, Università degli Studi di Milano, via Giuseppe Colombo 71, 20133 Milano, Italy; gianpaolo.tornatore@email.it (G.T.); francesca.caccavale@studenti.unimi.it (F.C.); stefano.longo@unimi.it (S.L.); fabio.esposito@unimi.it (F.E.); emiliano.ce@unimi.it (E.C.); 2 IRCCS Galeazzi Orthopaedic Institute, 20122 Milano, Italy

**Keywords:** EMG, quadriceps, gluteus maximus, adductor longus, weight training, strength training, front squat, back squat, feet stance

## Abstract

The present study investigated the activation of gluteal, thigh, and lower back muscles in different squat variations. Ten male competitive bodybuilders perform back-squat at full (full-BS) or parallel (parallel-BS) depth, using large feet-stance (sumo-BS), and enhancing the feet external rotation (external-rotated-sumo-BS) and front-squat (FS) at 80% 1-RM. The normalized surface electromyographic root-mean-square (sEMG RMS) amplitude of *gluteus maximus, gluteus medius, rectus femoris, vastus lateralis, vastus medialis, adductor longus, longissimus,* and *iliocostalis* was recorded during both the ascending and descending phase of each exercise. During the descending phase, greater sEMG RMS amplitude of *gluteus maximus* and *gluteus medius* was found in FS vs. all other exercises (*p* < 0.05). Additionally, FS elicited *iliocostalis* more than all other exercises. During the ascending phase, both sumo-BS and external-rotated-sumo-BS showed greater *vastus lateralis* and *adductor longus* activation compared to all other exercises (*p* < 0.05). Moreover, *rectus femoris* activation was greater in FS compared to full-BS (*p* < 0.05). No between-exercise difference was found in *vastus medialis* and *longissimus* showed no between-exercise difference. FS needs more backward stabilization during the descending phase. Larger feet-stance increases thigh muscles activity, possibly because of their longer length. These findings show how bodybuilders uniquely recruit muscles when performing different squat variations.

## 1. Introduction

The squat is one of the most popular exercises used to elicit lower-limb strength, hypertrophy, and power [1,2,3]. It consists of a simultaneous flexion-extension of the hip, knee, and ankle joints, with the important role of the lower back muscles that stabilize the upper body, and consequently the whole movement [4,5]. Particularly, the gluteal, thigh, and lower back muscles are strongly activated during both the ascending and descending phase [6,7,8,9].

The squat can be performed in a multitude of variations, depending for example on the place where the barbell is located, the squatting depth, the feet stance, and/or feet rotation. Consequently, we may have back-squat (BS) or front-squat (FS) when the barbell is placed over the shoulders or in front of the clavicular line, respectively [10]. Alternatively, the squatting depth may lead to parallel or full squat, where the descending phase ends when the thighs are parallel to the ground or below this line, respectively [7]. Furthermore, the feet stance may be regular or wide, leading in the latter case to a so-called sumo-squat, where the direction of the feet can be parallel or rotated externally [11]. Obviously, all these independent parameters can be miscellaneously used to create many combinations of squatting techniques and exercises. Among these, full-BS, parallel-BS, FS, sumo-BS, and external-rotated-sumo-BS are widely performed in practice.

A number of studies have investigated the difference in muscle activation when different squat variations are performed. Overall, squatting depth was shown to affect *gluteus maximus* activation with inconsistent results, with greater activation recorded in partial vs. full squat performed by young resistance-trained men [12], greater activation in full vs. partial performed by experienced lifters [7], or no difference when performed by resistance-trained women [13]. Additionally, quadriceps activation was overall greater in full vs. partial squat [14]. Interestingly, no difference in muscle activation was found comparing BS vs. FS performed with 70% 1-RM by healthy men [10], while larger stance specifically activates medial thigh muscles in experienced lifters [15], although no difference was found in gluteal muscles activation [11].

Bodybuilders have a unique capacity to perform exercises with a profound consistency of their technique, and were recently used to investigate the differences in muscle activation when bench press [16] or shoulder raise variations [17] are performed. Additionally, examining the muscle activation during both the concentric and the descending phase may help practitioners to characterize the strength and the hypertrophic stimuli, given both the short-term [18,19] and long-term unique responses following traditional or eccentric-based exercise training [2,20,21,22]. Therefore, the present study investigated the differences in the gluteal, thigh, and lower back muscles’ activation in bodybuilders when varying the squatting technique. Particularly, the exercises selected were full-BS, parallel-BS, sumo-BS, external-rotated-sumo-BS, and FS, and the gluteal, thigh, and lower back muscles’ activation were recorded during both the ascending and descending phase.

## 2. Materials and Methods

### 2.1. Study Design

The present investigation was designed as a cross-over, repeated-measures, within-subject study. The participants were involved in seven different sessions. In the first five sessions, the 1-RM was measured in full-BS, parallel-BS, sumo-BS, external-rotation-sumo-BS, and FS in random order. In the sixth session, the participants were familiarized with the selected loads and the electrodes placement. In the seventh session, the muscles’ maximum activation was first measured, i.e., the activation during a maximum voluntary contraction. Then, after a minimum of 30 min of passive recovery, the participant performed a non-exhausting set for each exercise performed in a random order, with an inter-set pause of 10 min. Each session was separated by at least three days, and the participants were instructed to avoid any further form of resistance training for the entire duration of the investigation.

### 2.2. Participants

The present investigation was advertised by the investigators during some regional and national competitions, and to be included in the study, the participants had to compete in regional competitions for a minimum of 5 years. Additionally, they had to be clinically healthy, without any reported history of upper-limb and lower back muscle injury and neurological or cardiovascular disease in the previous 12 months. To avoid possible confounding factors, the participants competed in the same weight category (Men’s Classic Bodybuilding <80 kg, <1.70 m), according to the International Federation of Body Building Pro-League. The use of drugs or steroids was continuously monitored by a dedicated authority under its regulations, although we could have not checked for it. Thereafter, 10 male competitive bodybuilders (age 29.8 ± 3.0 years; body mass 77.9 ± 1.0 kg; stature 1.68 ± 0.01 m; training seniority 10.6 ± 1.8 years) were recruited for the present procedures. The participants were asked to abstain from alcohol, caffeine, or similar beverages in the 24 h preceding the test. After a full explanation of the aims of the study and the experimental procedures, the participants signed a written informed consent. They were also free to withdraw at any time. The current design was approved by the Ethical Committee of the Università degli Studi di Milano (CE 27/17) and performed following the Declaration of Helsinki (1975) for studies involving human subjects.

### 2.3. Maximum Voluntary Isometric Activation

The maximum voluntary isometric activation of gluteus maximus, gluteus medius, rectus femoris, vastus lateralis, vastus medialis, erector spinae longissimus, and erector spinae iliocostal was assessed in random order. The electrodes were placed on the dominant limb, defined as the one preferred to kick a ball [2]. The participants were required to exert their maximum force against manual resistance. Each attempt lasted 5 s and three attempts were completed for each movement separated by 3 min of passive recovery [16,17]. The operators provided strong standardized verbal encouragements to push as hard as possible against the resistance exerted. The surface electromyography (sEMG) electrodes were placed following the SENIAM recommendations [23]. To check for appropriate electrodes placement previous procedures were followed [17]. For example, if the electrode shifted over the innervation zone during part of the movement, the EMG amplitude was underestimated. Therefore, to check for any consequence due to a possible shift of the surface electrode over the innervation zone, a Fast-Fourier Transform approach was used, as suggested in a previous investigation [24]. Briefly, the electrode placement on each muscle was checked during the warm-up phase of each exercise, analyzing the power spectrum profile of the sEMG signal recorded at the starting-, middle-, and endpoint of each exercise in all muscles. The correct electrode placement results in a typical belly-shaped power spectrum profile of the EMG signal, while noise, motion artifacts, power lines, and electrodes placed on the innervation zone or myotendinous junction generate a different power spectrum profile [24]. If the power spectrum did not match with the typical belly-shaped power spectrum profile in any of the temporal points, the electrodes were repositioned, and the procedures repeated so to have a clear EMG signal from all the muscles throughout the movement. The same experienced operator placed the electrodes and checked the power-spectrum profile. This approach was shown to provide very high reliability in sEMG data [16,17].

For *gluteus maximus*, the participants laid prone with the flexed knee and the electrode was placed below the line between the posterior-superior iliac spine and the trochanter major [13]. The participants were then asked to extend the hip against a manual resistance on the distal thigh [13]. For *gluteus medius*, the participant laid on a side and the electrodes were placed at 50% on the line from the crista iliaca to the trochanter. The participant was then asked to abduct the limb against manual resistance [23]. For *rectus femoris*, *vastus lateralis,* and *vastus medialis,* the participants sat on a table with the knees in slight flexion and the trunk slightly bent backward. The electrode were respectively placed at 50% and 2/3 on the line between the anterior-superior iliac spine and the lateral side of the patella and at 90% on the line between the anterior-superior iliac spine and the joint space in front of the anterior border of the medial ligament [23]. The participant were then asked to extend the knee against manual resistance [23]. The *adductor longus* belly was found midway between the origin at the pubic tubercle and the insertion at the medial linea aspera of the femur [25]. To ensure electrode placement, the test leg was passively abducted and the adductor longus muscle belly was palpated just distal to the muscle’s tendon, traced from the pubic tubercle on the medial side of the leg, and the participant was then asked to actively adduct the leg against resistance [25]. For *erector spinae longissimus* and *iliocostalis*, the participant laid prone and the electrodes were respectively placed at 2-finger width lateral from the processus spinalis of L1 and 1-finger width medial from the line from the posterior-superior iliac spine to the lowest point of the lower rib, at the level of L2 [23]. The participant was then asked to extend the trunk against manual resistance [23].

The electrodes were equipped with a probe (probe mass: 8.5 g, BTS Inc., Milano, Italy) that permitted the detection and the transfer of the sEMG signal by wireless modality. sEMG signal was acquired at 1000 Hz, amplified (gain: 2000, impedance and the common rejection mode ratio of the equipment are >1015 Ω//0.2 pF and 60/10 Hz 92 dB, respectively), and driven to a wireless electromyographic system (FREEEMG 300, BTS Inc., Milano, Italy) that digitized (1000 Hz) and filtered (filter type: IV-order Butterworth filter; bandwidth: 10–500 Hz) the raw sEMG signals.

### 2.4. 1-RM Protocol

The squat 1-RM was assessed following previous procedures [26] using an Olympic bar (Vulcan Standard 20 kg, Vulcan Strength Training System, Charlotte, NC, USA). Briefly, after a standardized warm-up consisting of 30 weight-free squats, the 1-RM attempts started from 80% of the self-declared 1-RM and additional 5% or less was added until failure [27]. Each attempt was separated by at least 3 min of passive recovery. A standard time under tension (2 s for the ascending and descending phase, 0.5 for the isometric phase) was used and the participants had to lower the bar until the thighs were parallel to the ground. A metronome was used to pace the intended duty cycle and a camera was used to provide a feedback about the squatting technique and depth. Strong standardized encouragements were provided to the participants to maximally perform each trial.

### 2.5. Exercises’ Technique Description

**Figure 1:**
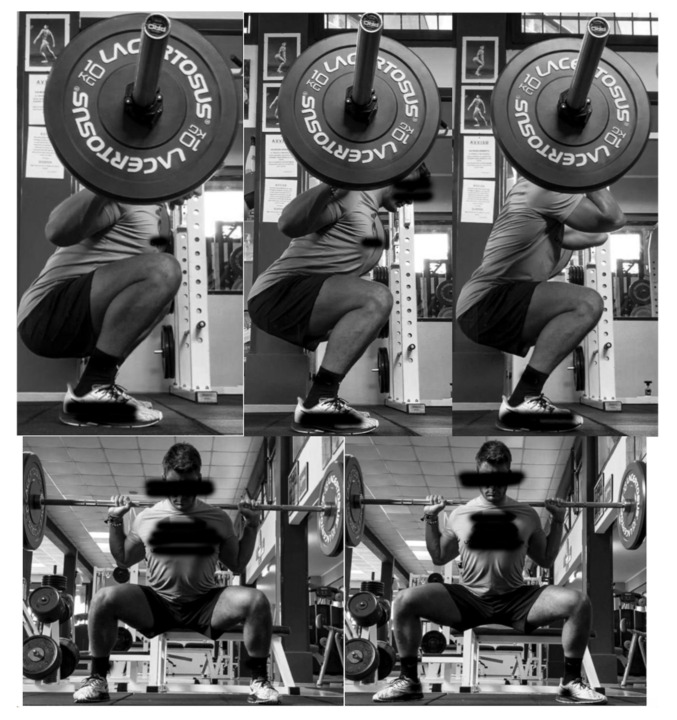
The squat variations are shown. From the left to the right, in the upper row: full-back squat (BS), parallel-BS, and front squat (FS). In the lower row: sumo-BS and external-rotated-sumo-BS.

The selected exercises are shown in Figure 1, and described here from left to right, first the upper and then the lower row. In parallel-BS, the bar was placed over the shoulder and the participants were required to descent until the thighs were parallel to the ground, with a regular feet stance. In full-BS, the bar was placed over the shoulder and the participants were required to descent below the parallel thighs, with a regular feet stance. In FS, the bar was placed in front of the clavicular line and sternum, and the participants were required to descent until the thighs were parallel to the ground, with a regular feet stance. In sumo-BS, the bar was placed over the shoulder, and the participants were required to descent until the thighs were parallel to the ground, with a two-fold feet stance compared to the previous exercises. In external-rotated-sumo-BS, the participants received the same instructions as for sumo-BS, with the exception of the feet that were rotated externally. Six non-exhaustive repetitions were performed for each exercise.

### 2.6. Data Analysis

The sEMG signals from both the peak value recorded during the maximum voluntary isometric activation and from the ascending and descending phases of each exercise were analyzed in time-domain, using a 25-ms mobile window for the computation of the root mean square (RMS). For the maximum voluntary isometric activation, the average of the RMS corresponding to the central 2 s was considered. During each exercise, the RMS was calculated and averaged over the 2 s of the ascending and descending phase. To identify the ascending and the descending phase, the sEMG was synchronized with an integrated camera (VixtaCam 30 Hz, BTS Inc., Milano, Italy) that provided the duration of each phase. Such a duration was used to mark the start and the end of each phase while analyzing the sEMG signal. The sEMG data were averaged excluding the first repetition of each set, to possibly have more consistent technique during the following repetitions. After, the sEMG RMS of each muscle during each exercise was normalized for its respective maximum voluntary isometric activation [16,17,27] and inserted into the data analysis.

### 2.7. Statistical Analysis

The statistical analysis was performed using a statistical software (SPSS 22.0, IBM, Armonk, NY, USA). The normality of data was checked using the Shapiro–Wilk test and all distributions were normal. Descriptive statistics are reported as mean (SD). The differences in the normalized EMG RMS were separately calculated for each exercise (5 levels) and phase (2 levels) using a two-way repeated-measures ANOVA. Multiple comparisons were adjusted using the Bonferroni’s correction. Significance was set at *p* < 0.05. The differences are reported as mean with 95% of confidence interval (95%CI). Cohen’s *d* effect size (ES) with 95% confidence interval (CI) was reported and interpreted according to the Hopkins’ recommendations: 0.00–0.19: trivial; 0.20–0.59: small: 0.60–1.19: moderate; 1.20–1.99: large; ≥2.00: very large [28].

## 3. Results

The 1-RM were as follows: 215(28) kg for full-BS, 238(31) kg for parallel-BS, 255(36) kg for sumo-BS, 258(41) kg for external-rotated-sumo-BS, and 176(33) kg for FS.

**Figure 2:**
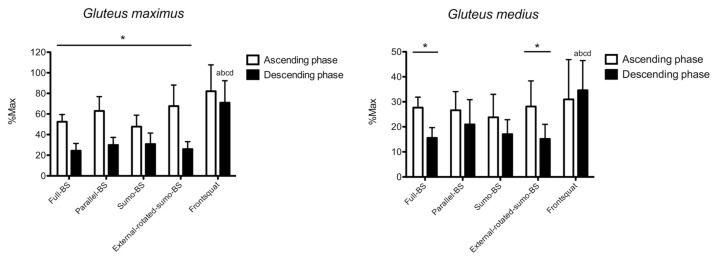
The surface electromyographic root-mean-square (sEMG) RMS amplitude of *gluteus maximus* and *gluteus medius* is shown. BS: back squat. *: *p* < 0.05 ascending vs. descending phase. a: *p* < 0.05 vs. full-BS. b: *p* < 0.05 vs. parallel-BS. c: *p* < 0.05 vs. sumo-BS. d: *p* < 0.05 vs. external-rotated-sumo-BS.

The results for *gluteus maximus* are shown in Figure 2. No phase x exercise interaction (*p* = 0.197) was found for the normalized RMS of *gluteus maximus*. A main effect was found for factor phase (*p* < 0.001), but not exercise (*p* = 0.097). With the exception of FS (11.1%, −6.5% to 28.8%, *p* = 0.11; ES: 0.48, −0.43 to 1.48), greater normalized RMS was found during the ascending vs. descending phase in all exercises (16.0% to 41.1%, *p* < 0.05; ES: 1.55 to 3.99). During the ascending phase, no between-exercise difference was observed. During the descending phase, greater normalized RMS was found in FS vs. full-BS (46.6%, 8.4% to 84.8%, *p* = 0.017; ES: 2.94, 1.58 to 4.05), parallel-BS (40.9%, 14.0% to 67.9%, *p* = 0.005; ES: 2.58, 1.31 to 3.63), sumo-BS (40.1%, 7.1% to 73.0%, *p* = 0.017; ES: 2.38, 1.16 to 3.41), and external-rotated-sumo-BS (44.9%, 10.3% to 79.5%, *p* = 0.012; ES: 2.83, 1.49 to 3.92).

The results for *gluteus medius* are shown in Figure 2. No phase x exercise interaction (*p* = 0.157) was found for the normalized RMS of *gluteus medius*. A main effect was found for factor phase (*p* = 0.002), but not exercise (*p* = 0.125). Greater normalized RMS was found during the ascending phase in full-BS (12.0%, 9.1% to 15%, *p* < 0.001; ES: 2.92, 1.56 to 4.02) and external-rotated-sumo-BS (12.9%, 4.2% to 21.7%, *p* = 0.010; ES: 1.57, 0.51 to 2.49). During the ascending phase, no between-exercise difference was observed. During the descending phase, greater normalized RMS was found in FS vs. full-BS (19.0%, 4.9% to 33.1%, *p* = 0.010; ES: 2.16, 0.98 to 3.16), parallel-BS (13.6%, 0.4% to 26.8%, *p* = 0.016; ES: 1.35, 0.10 to 2.70), sumo-BS (17.5%, 5.4% to 29.6%, *p* = 0.006; ES: 1.90, 0.78 to 2.86), and external-rotated-sumo-BS (19.4%, 7.3% to 31.5%, *p* = 0.003; ES: 2.10, 0.93 to 3.08).

**Figure 3:**
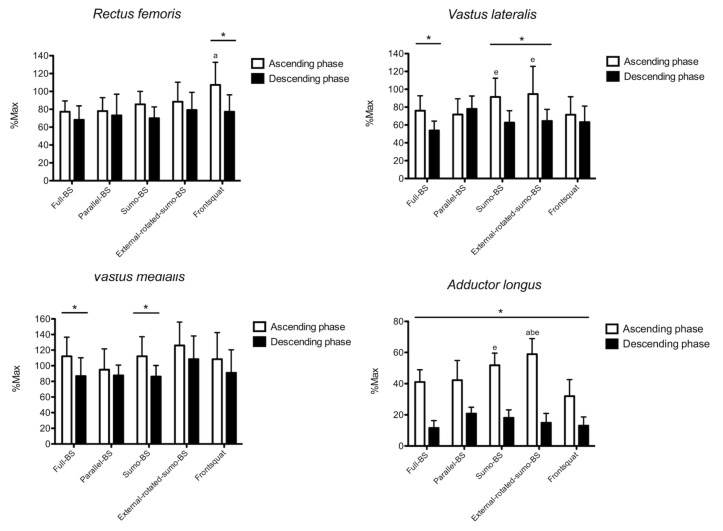
The surface electromyographic root-mean-square (sEMG) RMS amplitude of *rectus femoris, vastus lateralis, vastus medialis* and *adductor longus* is shown. BS: back squat. *: *p* < 0.05 ascending vs. descending phase. a: *p* < 0.05 vs. full-BS. b: *p* < 0.05 vs. parallel-BS. e: *p* < 0.05 vs. parallel front squat.

The results for *rectus femoris* are shown in Figure 3. Phase x exercise interaction (*p* = 0.038) was found for the normalized RMS, and no main effect was found for factor phase (*p* = 0.417) and exercise (*p* = 0.231). Greater normalized RMS was found during the ascending compared to the descending phase in FS (30.1%, 7.8% to 52.3%, *p* = 0.015; ES: 1.35, 0.33 to 2.25). During the ascending phase FS showed greater normalized RMS than full-BS (24.0%, 1.9% to 46.0%, *p* = 0.032; ES: 1.21, 0.21 to 2.11). No between-exercise difference was found during the descending phase.

The results for *vastus lateralis* are shown in Figure 3. Phase x exercise interaction (*p* = 0.026) was found for the normalized RMS, and a main effect was found for factor phase (*p* = 0.011), but not exercise (*p* = 0.457). Compared to the descending phase, greater normalized RMS was found during the ascending phase in full-BS (22.1%, 6.1% to 38.1%, *p* = 0.013; ES: 1.60, 0.54 to 2.53), sumo-BS (28.8%, 8.4% to 49.1%, *p* = 0.012; ES: 1.64, 0.57 to 2.58), and external-rotated-sumo-BS (30.0%, 14.6% to 45.5%, *p* = 0.002; ES: 1.26, 0.25 to 2.16). During the ascending phase, both sumo-BS (19.8%, 0.8% to 38.8%, *p* = 0.040; ES: 0.97, 0.01 to 1.85) and external-rotated-sumo-BS (23.0%, 3.8% to 42.1%, *p* = 0.019; ES: 0.88, −0.07 to 1.76) had greater normalized RMS than FS. No between-exercise difference was found during the descending phase.

The results for *vastus medialis* are shown in Figure 3. No phase x exercise interaction (*p* = 0.133) was found for the normalized RMS, and a main effect was found for factor phase (*p* < 0.001), but not exercise (*p* = 0.102). Compared to the descending phase, greater normalized RMS was found during the ascending phase in full-BS (25.2%, 11.8% to 38.5%, *p* = 0.003; ES: 1.06, 0.08 to 1.94) and sumo-BS (25.9%, 10.6% to 41.2%, *p* = 0.005; ES: 1.27, 0.26 to 2.17). No between-exercise difference was observed during both the ascending and descending phase.

The results for *adductor longus* are shown in Figure 3. Phase x exercise interaction (*p* = 0.032) was found for the normalized RMS, and a main effect was found for factor phase (*p* < 0.001) and exercise (*p* = 0.021). Compared to the descending phase, greater normalized RMS was observed during the ascending phase in all exercises (ES: 2.25 to 5.39). During the ascending phase, greater normalized RMS was found in external-rotated-sumo-BS than full-BS (17.9%, 1.7% to 34.0%, *p* = 0.029; ES: 2.01, 0.86 to 2.98), parallel-BS (16.7%, 3.0% to 30.3%, *p* = 0.017; ES: 1.47, 0.43 to 2.39), and FS [26.9%, 7.3% to 46.5%, *p* = 0.009; ES: 2.64, 1.35 to 3.70). Greater normalized RMS was also found for sumo-BS than FS (19.7%, 5.6% to 33.9%, *p* = 0.008; ES: 2.15, 0.98 to 3.14).

**Figure 4:**
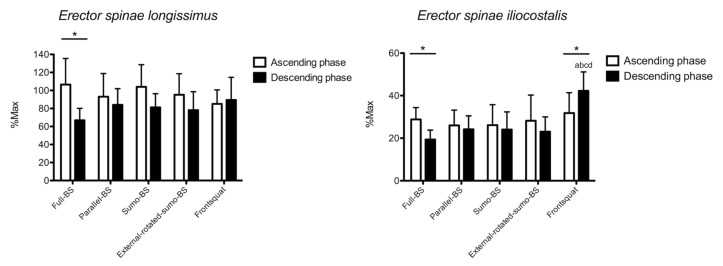
The surface electromyographic root-mean-square (sEMG) RMS amplitude of *erector spinae longissimus* and *erector spinae iliocostalis* is shown. BS: back squat. *: *p* < 0.05 ascending vs. descending phase. a: *p* < 0.05 vs. full-BS. b: *p* < 0.05 vs. parallel-BS. c: *p* < 0.05 vs. sumo-BS. d: *p* < 0.05 vs. external-rotated-sumo-BS.

The results for *erector spinae longissimus* are shown in Figure 4. Phase x exercise interaction (*p* = 0.004) was found for the normalized RMS, and a main effect was found for factor phase (*p* = 0.015), but not exercise (*p* = 0.477). Compared to the descending phase, greater normalized RMS was found during the ascending phase in full-BS (39.6%, 16.0% to 63.1%, *p* = 0.005; ES: 1.76, 0.67 to 2.71). No between-exercise difference was observed during both ascending and descending phase.

The results for *erector spinae iliocostalis* are shown in Figure 4. Phase x exercise interaction (*p* = 0.020) was found for the normalized RMS, and a main effect was found for factor exercise (*p* = 0.040), but not phase (*p* = 0.431). Compared to the descending phase, the normalized RMS was greater during the ascending phase in full-BS (9.4%, 4.5% to 14.3%, *p* = 0.003; ES: 1.91, 0.78 to 2.87) and lower in FS (−10.4%, −18.6 to −2.3, *p* = 0.019; ES: −1.14, −2.03 to −0.15). During the descending phase, FS showed greater normalized RMS than full-BS (22.9%, 14.6% to 31.2%, *p* < 0.001; ES: 3.29, 1.84 to 4.46), parallel-BS (18.1%, 0.5% to 35.7%, *p* = 0.043; ES: 2.37, 1.14 to 3.39), sumo-BS (18.2%, 12.1% to 24.3%, *p* < 0.001; ES: 2.14, 0.97 to 3.13), and external-rotated-sumo-BS (19.2%, 7.1% to 31.2%, *p* = 0.004; ES: 2.43, 1.19 to 3.46). No between-exercise difference was found during the ascending phase.

## 4. Discussion

The current study examined how different squat variations influence the activation of the main muscles involved in these exercises. Both *gluteus maximus* and *gluteus medius* were more active during the descending phase of FS compared to all other exercises. *Rectus femoris* was more active during the ascending phase of FS compared to full-BS compared to all other exercises, while no between-exercise difference was visible for *vastus medialis*. *Vastus lateralis* and *adductor longus* were more active during the ascending phase of sumo-BS and external-rotation-sumo-BS compared to all other exercises. Lastly, while no between-exercise difference was observed for *erector spinae longissimus*, *erector spinae iliocostalis* was more active during the descending phase of FS elicited compared to all other exercises. As such, varying the squatting technique seems to affect selectively the muscle activation.

### 4.1. Gluteal Muscles

FS showed very large increases in the *gluteus maximus* activation compared with all other exercises during the descending phase, with no between-exercise difference recorded during the ascending phase. A direct comparison with the literature is challenging, since few previous studies used similar design. When recording the sEMG RMS amplitude of *gluteus maximus* and distinguishing the ascending from the descending phase, no difference in FS vs. BS was found [29]. However, the load was maximal and performed by healthy men that limits the inference towards the present population. Additionally, we found that the *gluteus maximus* activation recorded here is much greater compared to the aforementioned study (e.g., 70% vs. 30% of the maximum activation during the descending phase of FS), which underlines the capacity of bodybuilders to increase muscle activation while training [30]. Moreover, no difference in gluteus maximus activation was found comparing FS, full-BS, and parallel-BS in trained women [13]. However, the authors did not specifically state which phase (ascending or descending or both) was examined, since it leads to argue that these findings are consistent with the no between-exercise difference recorded here during the ascending phase. Additionally, FS vs. BS was previously investigated, but no gluteal muscle was examined [10]. Lastly, the effect of stance does not seem to play a key role in *gluteus maximus* activation, which contrasts with the greater activation reported at greater stance [11,15]. Again, it is possible that the present bodybuilders population may have cancelled such a difference, since they were able to recruit the gluteus maximus more than just experienced lifters irrespectively of the stance. Similarly, *gluteus medius* resulted in greater activation during the descending phase of FS compared to all other exercise, with no between-exercise difference during the ascending phase. In a previous study, no difference in *gluteus medius* activation was observed when increasing the feet stance, confirming the present findings [11]. Taking all together, gluteal muscles seem to be particularly involved during the descending phase of FS. This may derive from the need to maintain an adequate trunk extension to avoid the barbell slipping forward (i.e., *gluteus maximus*), and to avoid a medial collapsing of the knees (i.e., *gluteus medius*), particularly when controlling the descending phase. As such, a frontal barbell placement seems to be a good option to increase the stimuli towards gluteal muscles while squatting.

### 4.2. Thigh Muscles

*Rectus femoris* showed greater activation in FS compared to full-BS during the ascending phase, with no other between-exercise differences. The lack of differences between full-BS and parallel-BS agrees with the no-difference found previously in powerlifters or weightlifters [31] or in healthy resistance-trained men [12]. Similarly with previous results, no difference in rectus femoris activation was reported when varying the squatting stance [11]. The reduced activation in full-BS vs. FS can be possibly explained by the greater *rectus femoris* length forced by the more vertical trunk in FS, which agrees with the greater work performed by the aforementioned gluteal muscles. Indeed, since *rectus femoris* acts as hip flexor, a more extended trunk corresponds to a longer length throughout the whole movement, thus increasing its activation as previously shown for deltoids [17] and triceps [32]. Both the sumo squats showed greater activation in *vastus lateralis* vs. FS. As suggested previously, larger stance makes hip and knee joint to exert more force to lift the load due to the non-favorable less vertical lever, thus increasing their recruitment [15]. Indeed, larger stance was shown to increase the *vastus lateralis* activation [33], rather than an external feet rotation alone, as previously reported [34]. Moreover, *vastus medialis* showed no between-exercise difference, with all exercises highly recruiting it. This may depend by the role of profound stabilizer of the patella across all movements, that enhances its activation when high loads have to be lifted. Lastly, larger stance and feet external rotation increased the *adductor longus* activation. This may depend on the need to stabilize the thigh position and keep the trajectory as vertical as possible in conjunction with the thigh external rotators, and on the longer muscle length at which adductor longus act at larger squat stance [11,15,35]. Taking together, larger feet stance may be used as an effective stimulus to increase the thigh muscles activity and could be implemented in the training practice accordingly.

### 4.3. Lower Back Muscles

*Erector spinae longissimus* showed no between-exercise difference, displaying a great activation across all exercises and during both the ascending and descending phase. In line with our results, no difference was found between BS and FS in experienced lifters [10], not even at different squatting depth in resistance trained men [12]. The study that investigated the effects of feet stance did not examine any lower back muscle [11,15,36], so a direct comparison cannot be made. However, given the high load and the consistent squatting technique, it is possible that the feet stance does not play a role in the *erector spinae longissimus* activation. Intriguingly, the activation of *erector spinae iliocostalis* was greater in FS compared with all other exercises during the descending phase. This may imply that FS needs additional balance control by mean of the trunk extensors to avoid any possible forward unbalancing. However, it should be noted that the net activation was much lesser than what observed in *longissimus,* meaning that the whole trunk and not only the lower back is involved in stabilizing the body. Lastly, both erectors’ activation was greater during the ascending vs. descending phase in full-BS. This may be accounted for the very closed joint angles that could need an additional backward action to start the movement from a non-favorable body position. In practice, in conjunction with the greater stimulus for the gluteal muscles, FS might be recommended to enhance the work of the lower back muscles.

### 4.4. Limitations

A number of limitations should be acknowledged. First, there is no information of any rear thigh muscle (e.g., *biceps femoris*) that could have deepened the between-exercise differences. Second, similarly, the stabilizer role of any anterior trunk muscle (e.g., *rectus abdominis*) was not examined. Third, we selected a group of squat variations among several possible different combinations, that cannot be examined in a single study, so further research is needed to widen these aspects. Fourth, adding kinematic data would deepen the knowledge and should be considered in future research. Last, it is acknowledged that the present results are specific for the present populations, and different sport background may result in different muscle activation.

## 5. Conclusions

In conclusion, the present study showed different muscle activation depending on the squat variation in competitive bodybuilders. A front vs. back bar position led to greater gluteal and lower back muscles activation compared to all other exercises. Additionally, larger feet stance increases the thigh muscles activation, particularly *rectus femoris*, *vastus lateralis,* and *adductor longus*. Lastly, squatting depth does not seem to promote any specific difference in muscle activation, with the exception of the greater *rectus femoris* activation in FS vs. full-BS. These findings could be used in resistance training practice to vary the training stimuli when performing the squat exercises depending on the muscle group needed to be highlighted. Additionally, the specific differences observed during the ascending or descending phase may increase the specificity of the training-induced effects.

## Data Availability

The data presented in this study are available on request from the corresponding author.
